# Role of fully-immersive virtual reality for paretic arm recovery in stroke rehabilitation

**DOI:** 10.3389/fneur.2025.1653749

**Published:** 2025-09-15

**Authors:** Seyoung Shin, Gyoseok Hwang, Yubin Kim, EunYoung Park, Dong Rae Cho, Hongsuk Baik, MinYoung Kim

**Affiliations:** ^1^Department of Rehabilitation Medicine, CHA Bundang Medical Center, CHA University School of Medicine, Seongnam, Republic of Korea; ^2^Digital Therapeutics Research Team, CHA Future Medicine Research Institute, Seongnam, Republic of Korea; ^3^Rehabilitation and Regeneration Research Center, CHA University School of Medicine, Seongnam, Republic of Korea

**Keywords:** Fugl–Meyer assessment, activities of daily living, neuroplasticity, occupational therapy, virtual reality

## Abstract

**Introduction:**

Rehabilitative training using fully immersive virtual reality (VR) has emerged as a promising method for improving motor function in patients with stroke. We evaluated the efficacy of VR technology as an adjunctive therapy in stroke rehabilitation.

**Methods:**

A VR program was designed by the research team to facilitate the movement of the paralyzed arm, and participation was offered to inpatients on a voluntary basis at a university-affiliated Rehabilitation Medicine Center. A retrospective analysis was conducted on patients with subacute stroke who were selected without bias between November 2015 and January 2020. Clinical outcomes were compared between the VR-received (*n* = 30) and the control groups (*n* = 14), which received only conventional rehabilitation. Outcome measures included the Fugl–Meyer Assessment for Upper Extremity, modified Barthel Index, Functional Independence Measure, Mini-Mental Status Examination, and Motor-Free Visual Perception Test, performed before and after each treatment, and changes in the scores between the two time points were measured. A subgroup analysis was conducted according to the number of VR interventions in the VR group. Multivariate regression was used to control for confounding variables.

**Results:**

There were no significant differences in baseline demographic characteristics and functional abilities between the two groups, except for a longer interval between the pre- and post-assessments in the control group (*p* < 0.01). The VR group showed a significantly greater improvement in Fugl–Meyer Assessment for Upper Extremity scores on the affected side than the control group did (*p* = 0.01). Multivariate analysis identified VR intervention as an independent predictor of motor improvement (*β* = 15.78), whereas delayed rehabilitation onset was negatively associated with recovery (*β* = −0.83).

**Conclusion:**

In this case-control study, the findings suggest that incorporating fully immersive VR-based training in rehabilitation may enhance upper limb function recovery in patients with stroke in the subacute phase. Further randomized controlled studies are required to confirm these findings.

## 1 Introduction

Stroke survivors often experience functional impairments across multiple domains, resulting in a high risk of severe disability and a marked reduction in their quality of life (1). Motor impairment is one of the most important disabilities after a stroke. Upper limb hemiparesis is particularly prevalent, affecting approximately 85% of patients with stroke; however, the recovery of upper limb function is slower and more challenging than that of lower limbs (2). Therefore, upper limb hemiparesis poses unique challenges in restoring fine motor skills essential for performing activities of daily living (ADL). To address these issues, novel clinical approaches have been introduced to enhance the upper motor function in patients with stroke.

The concept of the “golden time” in rehabilitation for patients with subacute stroke refers to the critical period shortly after a stroke when neuroplasticity is heightened, making rehabilitation potentially more effective (3). In conventional rehabilitation for the subacute phase of stroke, theories suggest that repetitive practice of functional movements facilitates recovery (4). However, repetitive exercises may diminish patient motivation and engagement. In this context, virtual reality (VR) training, which incorporates game-like elements, has gained attention owing to its potential to enhance patient participation (5, 6). Preliminary studies have reported that fully immersive VR applications are safe and well-received by patients because of their enjoyable and interesting nature (7). Moreover, several studies have reported that patients with stroke can benefit from rehabilitative training using VR, particularly by improving the performance of the affected upper limbs (8, 9). Previous meta-analyses regarding VR use for stroke reported positive outcomes in ADL with a standardized mean difference of 0.58 and significant gains in Fugl–Meyer Assessment (FMA) scores (5, 10).

In this study, we contribute to this emerging field by demonstrating the clinical feasibility and potential benefits of a fully immersive VR-based rehabilitation approach. A previous meta-analysis reported that VR rehabilitation was significantly associated with improvements in motor outcomes, particularly in hand and upper-extremity function tests (5). However, it is essential to explore the broader effects of VR on cognition, ADL, and visual perception when applied under a unified protocol in the same group of patients. Furthermore, considering that the FMA and Barthel Index include multiple subcategories, analyzing changes not only in total scores but also in individual subdomains could provide more detailed clinical insights.

In this study, we applied a fully immersive VR rehabilitation program in patients with subacute stroke and evaluated its effects across multiple functional domains, including motor function, cognition, ADL, and visual perception, with a detailed analysis of the subcategory scores. Furthermore, we investigated whether the number of VR therapy sessions influenced functional outcomes.

## 2 Materials and methods

### 2.1 Fully-immersive virtual reality protocol

**Figure 1:**
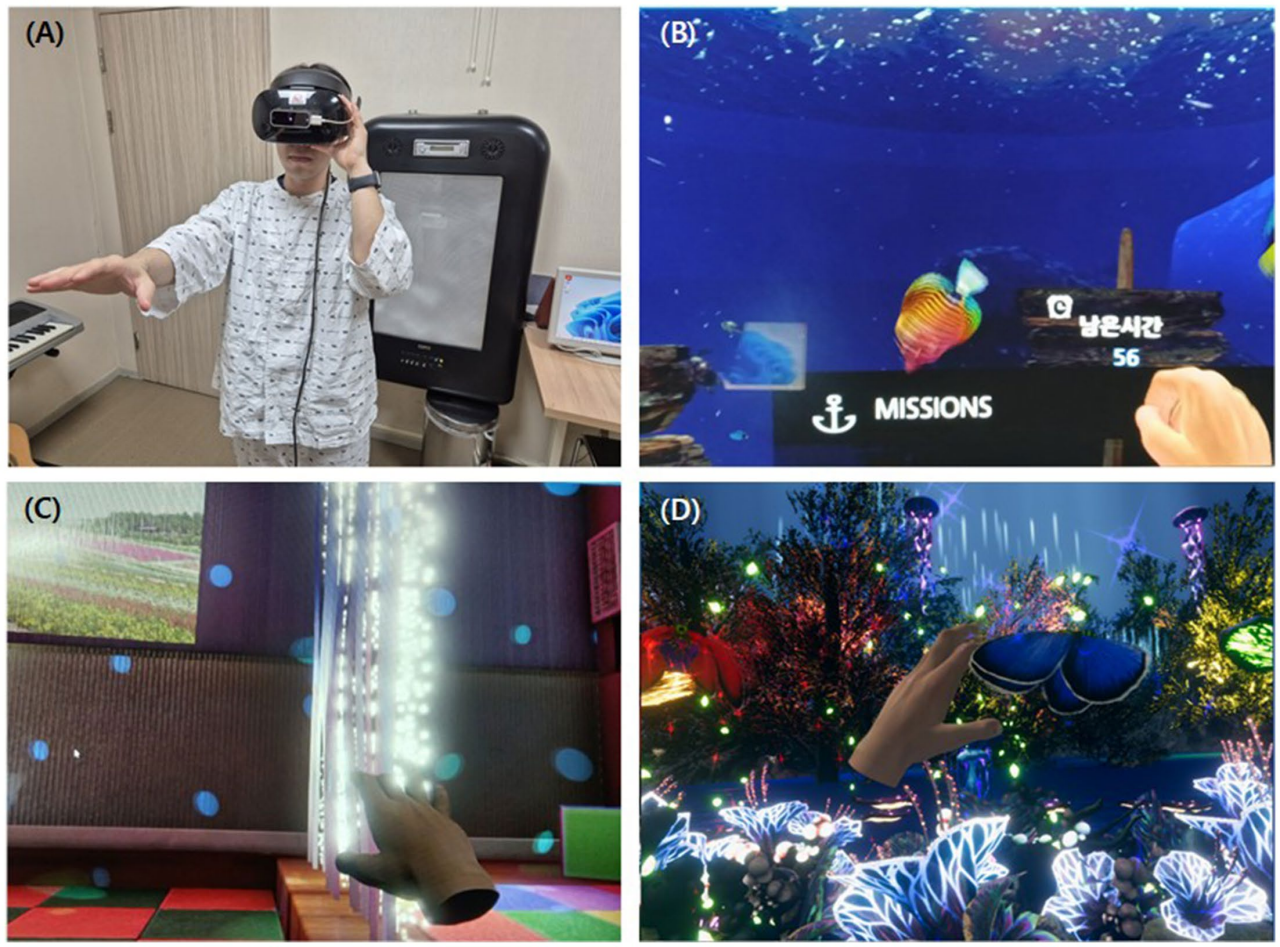
A display screen capture of virtual reality programs. **(A)** Actual usage of the VR device. **(B–D)** Contents of the VR program. **(B)** A display screen capture of VR training content, “Undersea Adventure.” **(C)** A display screen capture of VR training content, “VR Snoezelen.” **(D)** A display screen capture of VR training content, “Avatar.” VR, virtual reality.

The VR program used in this study was independently developed through close collaboration between clinical rehabilitation physicians from the CHA Bundang Medical Center and technology developers from Side9 Inc. (Republic of Korea). Three types of VR rehabilitation training content were developed using the Unity3D game engine (Figure 1). Patients could voluntarily participate in adjunctive fully immersive VR rehabilitation training, which involved wearing a VR headset device (Oculus Rift, United States), a motion sensor (HTC Vive tracker, Taiwan), and a hand-tracking device (LeapMotion, United States). The VR intervention consisted of three components: (1) “Undersea Adventure,” which promoted voluntary movement and attention by encouraging patients to catch fish in an underwater virtual environment while enhancing short-term memory; (2) “VR Snoezelen,” which provided multi-sensory stimulation (visual and auditory) designed to promote relaxation, reduce stress, and stimulate cognitive activity; and (3) “Avatar,” aimed at enhancing voluntary motor control and selective attention by catching color-coded butterflies in a dynamic forest setting.

Patients were offered voluntary participation in the VR program, with sessions conducted up to three times per week for 5–30 min per session, depending on individual rehabilitation schedules, for approximately a month. All sessions were supervised by a designated occupational therapist, who adjusted the difficulty level based on each patient’s functional status. Demographic and clinical data, including age, sex, stroke type (ischemic/hemorrhagic), side of hemiparesis, time since onset, and length of hospital stay, were collected retrospectively. This retrospective study was approved by the hospital’s institutional review board (Approval No. 2020-09-015).

### 2.2 Participants

This study is a retrospective case-control study. We included patients with functional impairment who were hospitalized at the Department of Rehabilitation, CHA Bundang Medical Center, South Korea, between November 2015 and January 2020. Inclusion criteria were as follows: (1) Underwent baseline and discharge rehabilitation assessments, including the Mini-Mental State Examination (MMSE), modified Barthel Index (MBI), Motor-Free Visual Perception Test (MVPT), functional independence measure (FIM), and FMA during admission (2) Age >18 years, (3) Diagnosis of ischemic or hemorrhagic stroke, (4) MMSE ≥10 with the ability to follow one-step commands, (5) Presence of definite hemiplegia or hemiparesis, (6) Stroke onset duration <1 month (or 40 days), and (7) length of hospital stay <1 month (or 40 days). Among the patients who met the inclusion criteria, we compared individuals who received occupational therapy (OT), including VR-based upper limb training at least three times, to a control group that received only conventional OT. VR participation was based on voluntary enrollment. As this was a retrospective case-control study, informed consent was not obtained from all participants.

### 2.3 Intervention

Both the VR and control groups received our routine rehabilitation program, which consisted of physical therapy (PT) focusing on gait and general muscle strengthening for 1 h twice daily, 5 days a week, with one extra session on Saturdays. OT was aimed at improving upper limb function and ADL ability which was provided with the same weekly schedule. On weekdays, the control group received two sessions of 30-min conventional OT sessions, whereas one of these sessions was replaced with a 30-min VR training session in the VR group. The frequency and duration of all the therapy sessions were the same as those in the control group. The number of VR sessions varied across patients, and a subgroup analysis was conducted by stratifying participants based on whether they received ≥10 sessions (upper quartile) versus <10 sessions. All the patients were also provided a self-exercise program to be performed independently during non-treatment periods.

### 2.4 Outcome measures

Functional outcome scores were obtained retrospectively from medical records, which were consistently collected before and after approximately 1 month of rehabilitation treatment. Specifically, our rehabilitation department routinely administered functional assessments for all inpatients upon admission and discharge, and these records were used in this study. We used the upper limb motor function scores of the FMA, MMSE, MBI, MVPT, and Cognitive/Motor components of the FIM. The FMA is a 100-point evaluation method used to assess the recovery of patients with motor dysfunction (11). It assesses the upper and lower extremity function. In this study, only the motor function of the upper limb (maximum score = 66) was analyzed to focus on upper limb movement recovery. The FMA score comprises four subcategories: A to assess function at the proximal joints, such as the shoulder, elbow, and forearm (maximum 32 points); B to evaluate function at the wrist level (maximum 10 points); C to assess hand function (maximum 14 points); and D to evaluate overall upper limb coordination and processing speed (maximum 6 points). The MMSE is a test to assess cognitive function. It is scored out of 30, with a score below 24 suggesting cognitive impairment (12). The MBI consists of 10 subcategories, each scored according to the patient’s level of independence (13). The total score ranges from 0 to 100, with higher scores indicating a patient’s ability to perform ADL. To assess visual perception, the MVPT, a 65-point evaluation method which consists of 36 items that are not affected by motor skills, was performed (14). The FIM is a 126-point evaluation method used to assess the patient’s ability to function independently and consists of two domains (15): the cognitive (maximum score = 35) and the motor (maximum score = 91) components. The motor domain has 13 items, and the cognitive domain has 5 items, each rated on a scale from 1 (totally dependent on assistance) to 7 (totally independent).

Licensed physical or occupational therapists, who also passed qualification tests for the measurements, performed the functional evaluations. According to the clinical research team’s protocol, the intra- and inter-rater reliabilities of the evaluator scoring were established, with correlation coefficients of 0.95.

### 2.5 Statistical analysis

Normality was confirmed using the Shapiro–Wilk test results. For comparisons between two independent groups, the Wilcoxon signed rank test or the independent samples *t*-test was used. For comparisons of three independent groups, the Kruskal–Wallis test or one-way analysis of variance was used, depending on normality. When analyzing categorical variables, the chi-square test was used to compare proportions. Finally, to adjust for potential confounders, a multivariable linear regression analysis was performed with ΔFMA as the dependent variable. The adjustment factors were selected by clinical insights, including age, sex, time since stroke onset, number of VR sessions. *p*-values <0.05 were considered statistically significant. All analyses were performed using R (version 4.0.3; R Foundation for Statistical Computing, Vienna, Austria) and GraphPad Prism (version 10.0; GraphPad Software, San Diego, CA, United States).

## 3 Results

**Figure 2:**
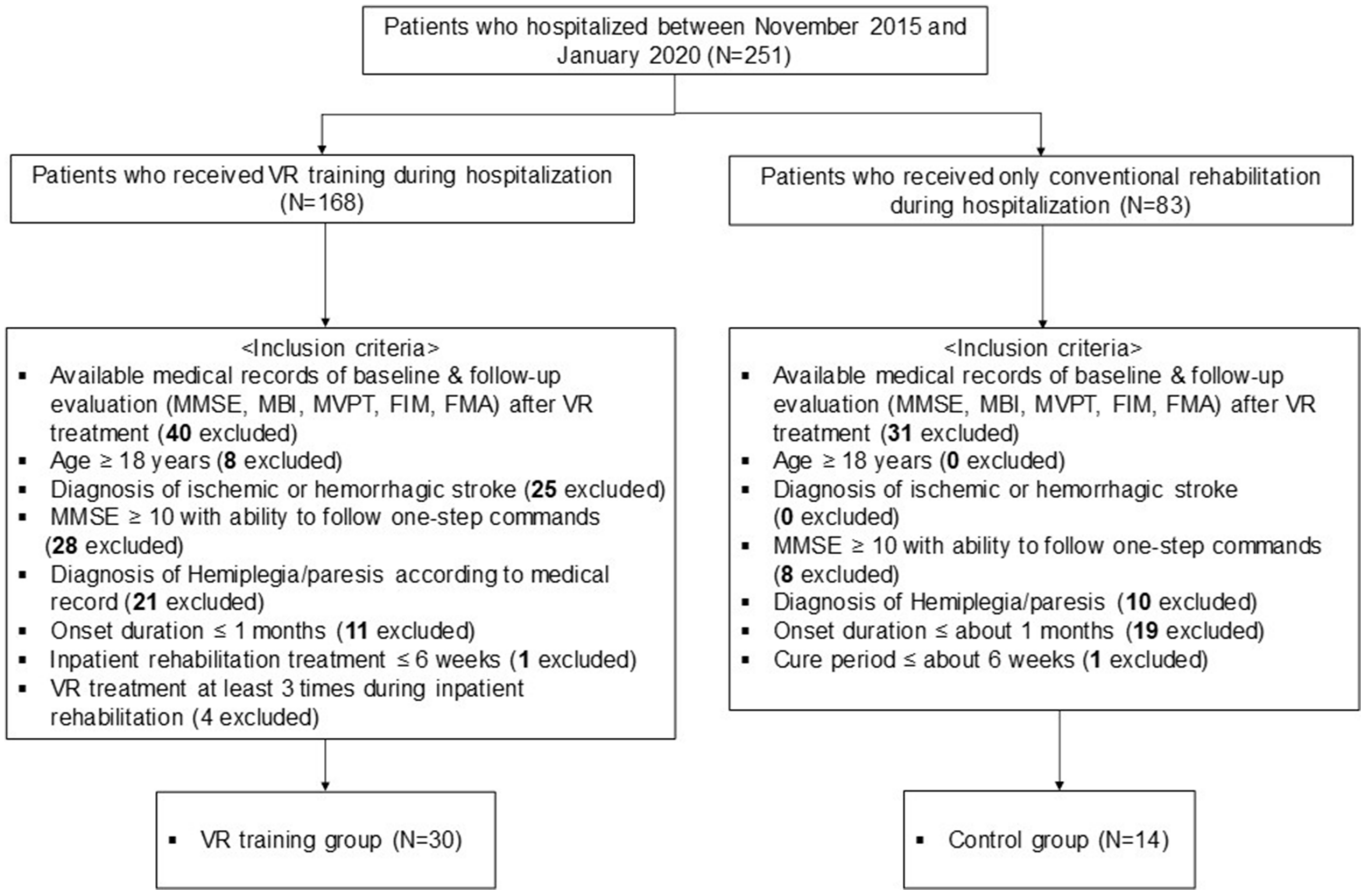
Flowchart of the study and dataset analyses. MMSE, Mini-Mental State Examination; MBI, modified Barthel Index; MVPT, Motor-Free Visual Perception Test; FIM, Functional Independence Measure; FMA, Fugl–Meyer Assessment.

Of the 251 patients admitted for functional rehabilitation between November 2015 and January 2020, 30 who underwent conventional rehabilitation with adjunctive VR therapy and 14 who underwent conventional rehabilitation alone met the inclusion criteria and were included in the final analysis (Figure 2). Baseline demographic and clinical characteristics were comparable between the two groups, except for the interval between the pre- and post-assessments, which was significantly longer in the control group (VR: 28.40 ± 3.31 days vs. control: 33.71 ± 4.84 days; *t* = 4.27, *p* < 0.01) (Table 1). Additionally, the VR group received a median of 6.00 (interquartile range: 4.00; 10.00) VR training sessions during the inpatient rehabilitation period. There were no significant baseline differences in functional assessment scores between the groups (Table 1).

**Table 1:** Demographic characteristics of the patients.

Variable	Total (*N* = 44)	VR (*N* = 30)	Control (*N* = 14)	Test statistic	*p-*value
Sex				*χ*^2^ < 0.001	1.00
Male	25 (56.82%)	17 (56.67%)	8 (57.14%)		
Female	19 (43.18%)	13 (43.33%)	6 (42.86%)		
Age	57.30 ± 14.90	55.27 ± 14.01	61.64 ± 16.32	*t* = 1.33	0.19
Days from stroke onset	19.60 ± 6.81	20.21 ± 6.54	18.14 ± 7.47	*t* = −0.98	0.33
DM	9 (20.45%)	9 (30.00%)	0 (0.0%)	*χ*^2^ = 3.60	0.06
HTN	17 (38.64%)	12 (40.00%)	5 (35.71%)	*χ*^2^ < 0.001	1.00
Type				*χ*^2^ = 3.04	0.08
Ischemic	28 (63.64%)	16 (53.33%)	12 (85.71%)		
Hemorrhagic	16 (36.36%)	14 (46.67%)	2 (14.29%)		
Hemiplegic side				*χ*^2^ = 0.28	0.60
Right	21 (47.73%)	13 (43.33%)	8 (57.14%)		
Left	23 (52.27%)	17 (56.67%)	6 (42.86%)		
Number of VR sessions (count)	4.00 [0.00; 6.50]	6.00 [4.00; 10.00]	—		—
Assessment interval (days)	30.09 ± 4.55	28.40 ± 3.31	33.71 ± 4.84	*t* = 4.27	<0.01^*^
Baseline functional assessments					
MMSE	25.00 [19.00; 27.00]	25.00 [19.00; 28.00]	24.00 [19.00; 27.00]	*W* = 183	0.50
MBI	52.73 ± 21.00	50.90 ± 20.14	56.64 ± 23.02	*t* = 0.84	0.41
MVPT	40.64 ± 14.01	39.97 ± 13.32	42.15 ± 15.91	*t* = 0.46	0.65
FIM cognition	28.00 [22.50; 32.00]	29.00 [25.00; 34.00]	25.50 [21.00; 30.00]	*W* = 150.5	0.18
FIM motor	48.49 ± 15.94	47.72 ± 15.00	50.07 ± 18.21	*t* = 0.45	0.66
FMA affected side	45.50 [23.00; 62.00]	39.00 [23.00; 55.00]	60.00 [23.00; 64.00]	*W* = 275	0.10

Test statistic values are presented as *t*, *W*, or *χ*^2^ depending on the test performed. *p*-values for differences in distribution were analyzed using the Wilcoxon signed-rank test or chi-square test, ^*^p < 0.05. VR, virtual reality; HTN, hypertension; DM, diabetes mellitus; MMSE, Mini-Mental State Examination; MBI, modified Barthel Index; MVPT, Motor-Free Visual Perception Test; FIM, Functional Independence Measure; FMA, Fugl–Meyer Assessment.

**Figure 3:**
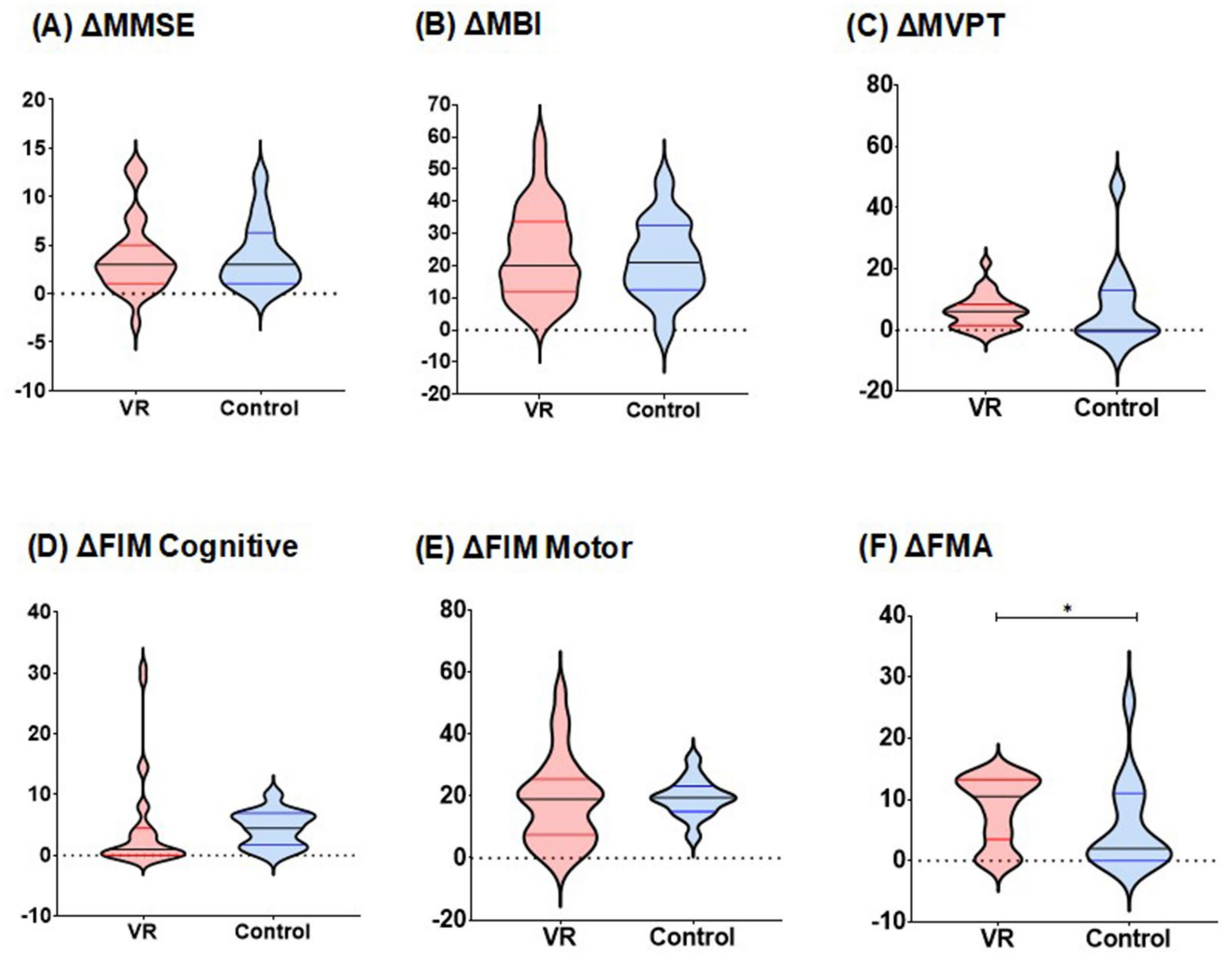
Comparison of changes in functional assessment scores between two groups. **(A)** Changes in MMSE scores during pre- and post-intervention. **(B)** Changes in MBI scores during pre- and post-intervention. **(C)** Changes in MVPT scores during pre- and post-intervention. **(D)** Changes in FIM cognitive scores during pre- and post-intervention. **(E)** Changes in FIM motor scores during pre- and post-intervention. **(F)** Changes in affected side FMA scores during pre- and post-intervention, *p*-values for differences in distribution were analyzed using the Wilcoxon signed rank test,^*^p < 0.05. MMSE, Mini-Mental State Examination; MBI, modified Barthel Index; MVPT, Motor-Free Visual Perception Test; FIM, Functional Independence Measure; FMA, Fugl–Meyer Assessment.

Figure 3 shows the between-group analysis of the VR and control groups regarding the score changes. The VR group exhibited significantly greater FMA score elevation in the affected upper limb (ΔFMA) than the control group did [VR: 15.50 (4.00; 30.00) vs. control: 2.00 (0.00; 11.00); *W* = 109.5, *p* = 0.01]. Detailed information on the differences in FMA total and subcategory scores for both groups is provided in Table 2. The VR group showed significantly greater improvement after the intervention in all subcategories of the FMA assessments (all *p* < 0.05).

**Table 2:** Comparison of subcategory scores of FMA between the two groups.

Variable	VR group (*N* = 30)	Control group (*N* = 14)	*W*	*p*-value
ΔFMA total	15.50 [4.00; 30.00]	2.00 [0.00; 11.00]	109.5	0.01^*^
FMA subcategory				
ΔShoulder/Elbow/Forearm	7.50 [1.00; 17.00]	1.00 [0.00; 4.00]	127	0.03^*^
ΔWrist	2.50 [0.00; 5.00]	0.00 [0.00; 1.00]	115	0.01^*^
ΔHand	2.00 [0.00; 6.00]	0.00 [0.00; 1.00]	103.5	0.01^*^
ΔCoordination/Speed	1.00 [0.00; 3.00]	0.00 [0.00; 1.00]	128.5	0.03^*^

Values are presented as the median [1st IQR; 3rd IQR] of the FMA scores on the affected side. *p*-values and test statistics *W* for differences in distribution were analyzed using the Wilcoxon signed rank, ^*^p < 0.05. VR, virtual reality; FMA, Fugl–Meyer Assessment; IQR, interquartile range.

For other functional outcomes, including ΔMMSE [VR: 3.00 (1.00; 5.00) vs. control: 3.00 (1.00;6.00); *W* = 183, *p* = 0.96], ΔMBI (VR: 24.03 ± 14.04 vs. control: 21.86 ± 12.10; *t* = 0.84, *p* = 0.62), ΔFIM cognition [VR: 1.00 (0.00; 4.00) vs. control 4.00 (2.00; 7.00); *W* = 150.5, *p* = 0.07], ΔFIM motor (VR: 18.34 ± 14.03 vs. control: 19.43 ± 6.12; *t* = 0.45, *p* = 0.73) and ΔMVPT [VR: 6.00 (2.00; 8.00) vs. control: 0.00 (0.00; 12.00); *W* = 275, *p* = 0.43], there were no statistically significant differences between the two groups.

A subgroup analysis based on the number of VR rehabilitation sessions is shown in Supplementary Table 1. Given that the third quartile of intervention sessions in the VR group was 10, we investigated the dose-dependent efficacy by comparing the group with intervention frequencies in the top 25% to the group with frequencies below this threshold. There were no significant differences in functional changes after intervention in all functional assessments between groups according to the VR session.

To avoid confounding effects due to small number of participants, a multivariable linear regression analysis was conducted (Table 3). The result confirmed that fully immersive VR intervention was an independent predictor of greater ΔFMA improvement (*β* = 15.78, SE = 6.48, *t* = 2.44, *p* = 0.02), even after adjusting for age, sex, time since stroke onset, number of VR sessions, and rehabilitation duration. And delayed initiation of rehabilitation appeared to negatively influence motor recovery (*β* = −0.83, SE = 0.30, *t* = −2.79, *p* = 0.01). Other covariates, including age (*t* = −1.79, *p* = 0.08), sex (*t* = 0.33, *p* = 0.75), VR time (*t* = −1.33, *p* = 0.19), and assessment interval days (*t* = −0.08, *p* = 0.93), were not significantly associated with ΔFMA.

**Table 3:** Multivariable linear regression analysis for predictors of upper limb motor improvement.

Variable	Estimate coefficient *β*	Standard error	*t*-value	*p*-value
VR intervention group (ref: control)	15.78	6.48	2.44	0.02^*^
Age	−0.27	0.15	−1.79	0.08
Sex	1.29	3.95	0.33	0.75
Days from stroke onset	−0.83	0.30	−2.79	0.01^*^
VR time	−0.48	0.36	−1.33	0.19
Assessment interval days	−0.05	0.57	−0.08	0.93

*β* as unstandardized regression coefficient, *t*-values and *p*-values are based on each coefficient in the multiple linear regression model with ΔFMA as the dependent variable. ^*^p < 0.05 was considered statistically significant. VR, virtual reality; FMA, Fugl–Meyer Assessment; SE, standard error.

## 4 Discussion

The results of this study suggest that adjunctive fully immersive VR rehabilitation may enhance upper limb motor recovery beyond that achieved with conventional rehabilitation alone in patients with subacute stroke. Notably, the VR group demonstrated a significantly greater improvement in upper limb motor function, as measured using the FMA, although the interval between the pre- and post-assessments was significantly longer in the control group, which may have allowed for a greater cumulative amount of conventional rehabilitation. Even after considering multiple confounders, VR intervention still independently related to the greater ΔFMA improvement. These findings imply that the goal-oriented nature of the VR intervention significantly facilitated upper-limb functional recovery.

Various types of VR rehabilitation systems have been developed to meet these clinical needs (16) and can be broadly categorized into fully immersive, semi-immersive, and non-immersive systems. Fully immersive VR refers to a VR system that completely blocks real-world perception, including visual, auditory, and haptic components. Our findings align with previous evidence that fully immersive VR systems may provide stronger sensorimotor stimulation and patient engagement than those with semi- or non-immersive VR systems. Semi-immersive and non-immersive systems are easier to implement; however, they provide less sensory engagement, potentially reducing user participation and the intensity of sensorimotor stimulation. Nevertheless, many studies in the field of stroke rehabilitation have used semi-immersive or non-immersive VR systems, focusing on their accessibility and ease of use (17, 18). A recent meta-analysis (5) reported only one study involving fully immersive VR, similar to ours, while 12 studies used semi-immersive VR (mainly screen- and motion-sensor-based systems), and 21 studies involved non-immersive VR (a simple virtual system operated through a screen, such as the Nintendo Wii). When using the FMA score as the outcome measure, VR interventions, particularly those employing immersive technologies, appeared to be effective in improving upper limb function. However, when only semi-immersive VR studies were analyzed, the improvement was not statistically significant. This suggests the need for more intervention-control studies focusing on fully immersive VR, which are currently limited. From this perspective, the current study adds clinically valuable significance.

A previous fully immersive VR randomized controlled trial with 65 participants showed significantly greater improvements in both FMA and FIM scores in the intervention group (19). Another randomized controlled trial using a novel fully immersive VR system showed significant improvements in the FMA score compared with that in the control; however, ADLs were not assessed in that study (20). Considering our study along with previous research, it is evident that fully immersive VR consistently plays a significant and positive role in improving upper limb function in patients with stroke. Despite the differences in the types of VR software and content used across studies, the fact that similar favorable outcomes were observed is encouraging and highlights the potential for fully immersive VR in stroke rehabilitation.

The proposed mechanisms underlying these improvements relate to repetitive visual, vestibular, and somatosensory feedback from VR rehabilitation, which activates neuronal firing, increases cortical reorganization, changes neural networks, and improves function (21). Given that the mechanism of VR rehabilitation is based on neuroplasticity (22), we expected that the group with more VR rehabilitation sessions would obtain significantly better functional outcome measures than those with fewer VR rehabilitation sessions. In this study, subgroup analysis did not demonstrate a clear dose-dependent effect of VR session number, which may be due to the small sample size. The neuroplasticity-driven benefits of VR rehabilitation may depend more on session quality and task-specific intensity rather than session number alone.

Virtual reality also offers scalability when used in combination with other neuromodulatory interventions such as non-invasive brain stimulation (NIBS). A recent meta-analysis reported that the combination of VR and NIBS led to significant improvements in upper limb motor function in stroke patients (23). Furthermore, as highlighted in a recent scoping review, immersive virtual reality holds promising potential for delivering intensive upper extremity rehabilitation within a telerehabilitation framework (24). These findings collectively underscore the potential of VR not only as a direct intervention but also as a platform adaptable to evolving multimodal and remote future rehabilitation strategies.

This study has some limitations. First, the retrospective design of the study may have introduced statistical confounding. In addition, voluntary participation in the VR group raises the possibility of self-selection bias, as more motivated or engaged patients may have opted into the intervention, potentially influencing outcomes. Moreover, the possibility of natural functional recovery cannot be ignored in patients with subacute stroke. Second, in terms of sample size and representativeness, some results may not be statistically significant because of low statistical power owing to the small sample size, thereby limiting generalizability. Given the limited sample size, the statistical power was insufficient to detect small to moderate effects, and the possibility of a type II error cannot be entirely ruled out.

Therefore, future prospective studies with larger sample sizes are warranted.

## 5 Conclusion

This study suggests that the use of fully immersive VR rehabilitation as an adjuvant to conventional rehabilitation in patients with hemiplegia or hemiparesis in the initial stages of subacute stroke may offer greater upper motor function improvements compared with using conventional rehabilitation alone, while the effect did not translate into improvements in ADL. Future prospective randomized controlled trials with larger sample sizes and standardized VR therapy protocols are warranted to validate the clinical efficacy and optimize the application of fully immersive VR rehabilitation in stroke care.

## Data Availability

De-identified data are available from the corresponding author upon reasonable request, subject to Institutional Review Board approval and a data-use agreement.
