# Supplementary material for: Role of fully-immersive virtual reality for paretic arm recovery in stroke rehabilitation
Source: Front Neurol. 2025 Sep 15;16:1653749. doi: 10.3389/fneur.2025.1653749 (PMC12477025; doi:10.3389/fneur.2025.1653749)
Supplement: Supplementary file 1 [file Table_1.docx]

Supplementary Material

# Supplementary Figures and Tables

## Supplementary Tables

**Supplementary Table 1. Functional differences according to VR intervention times**

|  | **VR < 10 times** | **VR ≥ 10 times** | **Test statistic** | ***P*-value** |
| --- | --- | --- | --- | --- |
|  | **(N=22)** | **(N=8)** |  |  |
| **Age** | 57.59 ± 13.18 | 48.88 ± 15.12 | t=1.54 | 0.13 |
| **Days from stroke onset** | 20.59 ± 6.44 | 19.75 ± 8.26 | t=0.29 | 0.77 |
| **VR time** | 5.00 [3.00; 6.00] | 19.00 [13.50; 20.50] | W=0 | <0.01^*^ |
| **Assessment interval (days)** | 27.32 ± 2.80 | 31.38 ± 2.83 | t=-3.50 | <0.01^*^ |
| **Functional changes after intervention** | | | | |
| ΔMMSE | 3.00 [1.00; 5.00] | 3.00 [2.00; 5.00] | W=86.5 | 0.96 |
| ΔMBI | 25.00 ± 15.56 | 21.38 ± 8.94 | t=0.62 | 0.54 |
| ΔMVPT | 6.00 [2.00; 9.00] | 5.50 [1.00; 7.50] | W=94.5 | 0.62 |
| ΔFIM cognition | 1.00 [0.00; 5.00] | 0.00 [0.00; 3.50] | W=107.5 | 0.25 |
| ΔFIM motor | 37.62 ± 13.25 | 21.75 ± 10.11 | t=-0.80 | 0.43 |
| ΔFMA affected side total | 18.00 [4.00; 30.00] | 9.00 [4.50; 25.00] | W=98.5 | 0.64 |
| ΔFMA Shoulder/Elbow/Forearm | 8.50 [2.00; 17.00] | 5.00 [0.50; 16.00] | W=94.5 | 0.78 |
| ΔFMA Wrist | 3.18 ± 2.89 | 2.00 [0.00; 4.50] | W=100 | 0.58 |
| ΔFMA Hand | 2.00 [0.00; 6.00] | 2.50 [0.00; 6.50] | W=93 | 0.83 |
| ΔFMA Coordination/Speed | 1.50 [0.00; 3.00] | 0.00 [0.00; 1.50] | W=125.5 | 0.07 |

*P*-values and test statistics W or t for differences in distribution were analyzed using the Wilcoxon signed rank or independent t-test, *^*^P*<0.05

Abbreviations: MMSE, Mini-Mental State Examination; MBI, modified Barthel Index; MVPT, Motor-Free Visual Perception Test; FIM, Functional Independence Measure; FMA, Fugl–Meyer Assessment; VR, virtual reality**.**
